# The Differential Effects of Auditory and Visual Stimuli on Learning, Retention and Reactivation of a Perceptual-Motor Temporal Sequence in Children With Developmental Coordination Disorder

**DOI:** 10.3389/fnhum.2021.616795

**Published:** 2021-03-31

**Authors:** Mélody Blais, Mélanie Jucla, Stéphanie Maziero, Jean-Michel Albaret, Yves Chaix, Jessica Tallet

**Affiliations:** ^1^Toulouse NeuroImaging Center, Université de Toulouse, Inserm, UPS, Toulouse, France; ^2^EuroMov Digital Health in Motion, Univ Montpellier, IMT Mines Ales, Montpellier, France; ^3^Octogone-Lordat, University of Toulouse, Toulouse, France; ^4^Hôpital des Enfants, Centre Hospitalier Universitaire de Toulouse, CHU Purpan, Toulouse, France

**Keywords:** procedural memory, rhythm, sensory modality, circular analyses, non-regular sequence, tapping

## Abstract

This study investigates the procedural learning, retention, and reactivation of temporal sensorimotor sequences in children with and without developmental coordination disorder (DCD). Twenty typically-developing (TD) children and 12 children with DCD took part in this study. The children were required to tap on a keyboard, synchronizing with auditory or visual stimuli presented as an isochronous temporal sequence, and practice non-isochronous temporal sequences to memorize them. Immediate and delayed retention of the audio-motor and visuo-motor non-isochronous sequences were tested by removing auditory or visual stimuli immediately after practice and after a delay of 2 h. A reactivation test involved reintroducing the auditory and visual stimuli after the delayed recall. Data were computed via circular analyses to obtain asynchrony, the stability of synchronization and errors (i.e., the number of supplementary taps). Firstly, an overall deficit in synchronization with both auditory and visual isochronous stimuli was observed in DCD children compared to TD children. During practice, further improvements (decrease in asynchrony and increase in stability) were found for the audio-motor non-isochronous sequence compared to the visuo-motor non-isochronous sequence in both TD children and children with DCD. However, a drastic increase in errors occurred in children with DCD during immediate retention as soon as the auditory stimuli were removed. Reintroducing auditory stimuli decreased errors in the audio-motor sequence for children with DCD. Such changes were not seen for the visuo-motor non-isochronous sequence, which was equally learned, retained and reactivated in DCD and TD children. All these results suggest that TD children benefit from both auditory and visual stimuli to memorize the sequence, whereas children with DCD seem to present a deficit in integrating an audio-motor sequence in their memory. The immediate effect of reactivation suggests a specific dependency on auditory information in DCD. Contrary to the audio-motor sequence, the visuo-motor sequence was both learned and retained in children with DCD. This suggests that visual stimuli could be the best information for memorizing a temporal sequence in DCD. All these results are discussed in terms of a specific audio-motor coupling deficit in DCD.

## Highlights

- General deficit in audio and visual motor synchronization with rhythmic stimuli in DCD.
- Auditory cueing improves learning and reactivation but not retention in DCD.
- Learning and retention of a visual sequence are preserved in DCD.

## Introduction

Perceptual-motor procedural leaning is the acquisition of new perceptual-motor skills (a series of simple or complex movement elements) with practice, and procedural learning tasks are numerous (Doyon and Benali, 2005; Doyon, 2008). Even if procedural learning of temporal sequences (with no spatial component) has been subject to fewer studies than spatio-temporal sequences (with a low temporal component, as in the traditional Serial Reaction Time Task), both kinds of learning involve learning the order of a repeated sequence (of spatial and/or temporal parameters, see Shin and Ivry, 2002). During practice, participants learn to predict the location and/or time of the subsequent stimulus, thus becoming faster to respond or synchronize with the stimuli. Temporal sequence learning is typically found in music training. For example, at the very beginning of training for drumming and the basis of rhythm in music, temporal sequences are practiced with low spatial parameters (only one drum and one drumstick). In this case, learning requires perceiving sensory input items, memorizing them in a structured temporal sequence through repetitive practice, retaining the sequence for a certain period and then retrieving this temporal sequence so as to recall it (Patel, 2003; Konoike et al., 2012, 2015).

Experimentally, the production of time intervals can be assessed via sensori-motor synchronization (SMS), which is synchronization of a motor output with a sensory stimulus (Fraisse, 1948; Fraisse et al., 1958; Repp et al., 2011; Repp, 2005; Repp and Su, 2013). Several studies have investigated SMS with isochronous stimuli, i.e., with identical time intervals between two stimuli (Jäncke et al., 2000; Chen et al., 2002; Pollok et al., 2009; Blais et al., 2014, 2015). Studies using an SMS paradigm in healthy adults highlight that synchronization with auditory stimuli is stable (Sowiński and Dalla Bella, 2013). Moreover, the literature shows that SMS depends on the sensory modality of the stimuli. When participants are required to tap with their index finger in synchronization with tones (auditory sequence) or flashes (visual sequence), SMS with an auditory stimulus is more accurate and stable than SMS with a visual stimulus (Fraisse, 1948; Semjen and Ivry, 2001; Chen et al., 2002; Repp and Penel, 2002; Patel et al., 2005; Tierney and Kraus, 2013; Blais et al., 2014, 2015). This suggests that rhythmic movements tend to synchronize with auditory more than visual rhythms (Repp and Penel, 2004; Kato and Konishi, 2006). Moreover, SMS is less accurate and stable with non-isochronous stimuli, i.e., a sequence with different time intervals between two stimuli (Patel et al., 2005; Andreou et al., 2015). Therefore, learning is required to achieve synchronization with non-isochronous (auditory or visual) stimuli, which involves alternating short and long delays between consecutive stimuli.

Regarding developmental coordination disorder (DCD), many studies have found evidence of impaired sensorimotor timing, and especially SMS, irrespective of the modality of the stimuli (auditory or visual stimuli) and the type of response (unimanual, bimanual, or verbal) (Volman and Geuze, 1998; Volman et al., 2006; de Castelnau et al., 2007, 2008; Whitall et al., 2008; Debrabant et al., 2013; Blais et al., 2017; Puyjarinet et al., 2017; Blais et al., 2018; Trainor et al., 2018; Lê et al., 2020). However, a recent hypothesis was postulated by Trainor et al. (2018) that one core deficit of DCD could be a specific auditory timing deficit. This deficit would lead to specific impairment of audio-motor synchronization in DCD compared to typical development.

Regarding learning, the model by Nicolson and Fawcett (2007) predicts that procedural learning would be altered in children with DCD because of a dysfunction of the cortico-striato-thalamo-cortical network. However, studies investigating this issue in DCD have reported inconsistent results (Wilson et al., 2003; Gheysen et al., 2011; Lejeune et al., 2013; Blais et al., 2018; Lê et al., 2020). Very recently, a specific deficit in learning and the retention of an auditory temporal non-isochronous sequence using verbal responses were found in DCD (Lê et al., 2020). The deficit was less apparent for learning and the retention of a visual temporal non-isochronous sequence. On the contrary, controlling temporal parameters with visual stimuli seems to be less affected and repeated practice allows learning and retention of the visual temporal non-isochronous sequence in DCD and typically-developing (TD) children equally. These results highlight that DCD children seem to present with an alteration in audio-verbal coupling that is not reduced despite repeated practice. This is in line with the hypothesis of Trainor et al. (2018).

Special emphasis is placed on dynamic changes in memory after learning. The memory dynamic corresponds to the retention and reactivation processes (Tallet, 2012; de Beukelaar et al., 2016; Fogel et al., 2017). The retention process is evaluated by recall tests without stimuli (immediately after practice and after a time delay) and reactivation is evaluated via reintroduction of the stimuli further to retention. Withdrawal of the stimuli during recall tests may reveal a persistence or forgetting of the memory trace and reintroduction of the stimuli may reactivate the memory trace having forgotten it. Therefore, in the present study, participants were required to practice temporal non-isochronous sequences by tapping on a keyboard in synchrony with auditory or visual stimuli. Afterward, they had to recall the sequences immediately after practice and recall again after a delay of 2 h. During the reactivation test, after the delayed recall (DEL), they were required to reproduce the sequence with the auditory or visual stimuli.

On this basis, this study aims to test the hypothesis for a specific audio-motor coupling impairment using manual responses in DCD when learning, retaining, and reactivating a new temporal sequence presented with either auditory or visual stimuli. In accordance with the hypothesis of an auditory timing deficit (Trainor et al., 2018), we expected that, compared to TD children, DCD children would have a deficit in SMS, learning, retention, and reactivation for a new audio-motor temporal sequence compared to a new visuo-motor temporal sequence. More operationally, we expected children with DCD to demonstrate a lesser decrease in mean asynchrony (and a lesser increase in stability) when practicing the audio-motor sequence compared to the visuo-motor temporal sequence. Moreover, for retention and reactivation, we expected that children with DCD would have a higher increase in asynchrony (and a lower increase in stability) for the audio-motor sequence compared to the visuo-motor sequence. In contrast, TD children were expected to have a higher increase in asynchrony (and a lower increase in stability) for the visuo-motor sequence compared to the audio-motor sequence.

## Materials and Methods

### Participants

Twelve children with DCD and 20 TD children aged 8–12 years took part in this study. They were all right-handed, as assessed by the 10-question version of the Edinburgh Handedness Inventory (Oldfield, 1971; mean laterality quotient: 88.77 ± 20.33; range: 20–100). Seven more children were examined for this study, but their MABC score did not meet the inclusion criteria of Movement Assessment Battery for Children (M-ABC) <5th percentile, so they were not included in the study. We did not include children with musical skills (more than 4 h a week of formal practice for over 1 year). Participants had corrected-to-normal vision and hearing, as verified by a pre-experimental questionnaire. The children were enrolled in the DYSTAC-MAP study (ANR-13-APPR-0010). Eleven DCD and 18 TD children who passed an MRI participated in the study by Lê et al. (2020).

The inclusion criteria for the DCD group were: (1) no attention deficit/hyperactivity disorder according to DSM-5 (American Psychiatric Association, 2013); (2) diagnosis of DCD by a pediatrician; and (3) a total impairment score in the M-ABC (Henderson and Sugden, 1992; Soppelsa and Albaret, 2004) lower than the 5th percentile. The TD group was included with a total score higher than or equal to the 15th percentile. All children were clinically screened for neurodevelopmental disorder according to the DSM5 criteria. Children with comorbidities including ADHD, specific language impairment and developmental dyslexia were excluded from both groups. Moreover, the children did not have any clinical signs of verbal dyspraxia. None of the children had an intellectual disability, which was assessed via two sub-tests of the Wechsler Intelligence Scale for Children, 4th version (Similarities and Picture Concepts; Wechsler, 2005). The protocol was promoted by the French Ethical Committee of the Institute for Medical Research (Inserm, 2014-AO1239-38).

The participant characteristics are given in Table 1.

**TABLE 1:** Motor and IQ assessment in both groups.

	TD (*n* = 20; 10 girls)		DCD (*n* = 12; 4 girls)		t(30)	p
		
	*M*	SD	*M*	SD		
Age (years)	10.17	1.30	9.63	1.18	1.59	0.12
M-ABC percentile	50.57	25.84	1.36	1.70	7.39	3.09.10^–8^
WISC-IV – SIM	12.7	2.93	12.25	3.81	0.92	0.36
WISC IV – PC	10.15	2.05	9.41	1.92	1.44	0.15

*M, mean; SD, standard deviation; M-ABC, Movement Assessment Battery for Children; WISC-IV, Wechsler Intelligence Scale for Children; SIM, Similitaries; PC, Picture Concepts.*

### Materials

In the experiment, a computer with Presentation software (Version 18.0, Neurobehavioral Systems, Inc., Berkeley, CA, United States^1^) was placed in front of the experimenter. This computer gave visual instructions and visual stimuli to a connected 24″ screen, located 80 cm from the participants. Auditory stimuli were sent through headphones.

The participant’s responses were collected via the same software using the key of the computer keyboard in front of him/her. The keyboard was connected to the computer via a USB port. The Presentation software recorded every time a key was pressed, which allowed recording with time precision in the tenths-of-milliseconds range. We ensured that the mean and stability for the uncertainties were very low and stable.

### Task

#### Control Task: Synchronization With Isochronous Sequence

All the children had to synchronize with a sequence of 10 stimuli appearing at an isochronous interval of 1 s, by tapping the key of the keyboard with the right index finger.

Two modalities were tested: auditory stimuli were given via short tones (100-ms duration, 500 Hz) through headphones and visual stimuli were given via yellow squares (100-ms duration), which appeared in the center of the computer screen.

#### Experimental Task: Learning to Synchronize With Non-isochronous Sequences

##### Practice (with stimuli)

**FIGURE 1:**
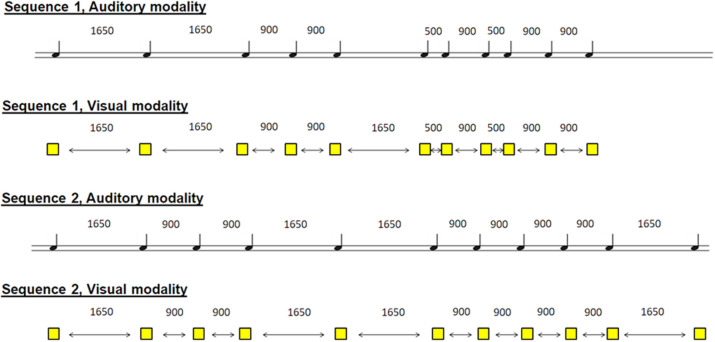
Schematic representation of the 4 sequences. 2 sequences in the auditory modality and 2 sequences in the visual modality. The participant had to learn one sequence in one modality and the other sequence in the other modality. Sequences and modalities were presented randomly among participants.

The participants were asked to learn two non-isochronous sequences by tapping the right index finger on the key in synchrony with auditory stimuli (one sequence) and visual stimuli (another sequence). The two sequences were a series of 11 stimuli, which appeared at non-isochronous intervals. The auditory sequence included 11 brief sounds (100-ms duration, 500 Hz) and came through headphones, and the visual modality was in the form of 11 yellow squares (100-ms duration), which appeared in the center of the computer screen. The inter-stimulus interval varied between 500, 900, and 1,650 ms within each sequence in a pre-established pseudo-randomized order (Figure 1). Please note that the sequences were presented in a counterbalanced way, so that the results could be interpreted with respect to the duration of the sequences.

The children had to learn two non-isochronous sequences (auditory and visual) (Figure 1). The participants were warned that they had to reproduce the sequence without stimuli (without a metronome) at the end of the practice [immediate recall (IMM)] and 2 h after the practice without stimuli (DEL) and then with stimuli (reactivation).

##### Immediate and delayed recall (without stimuli)

In these tasks, the children had to recall sequences by tapping the key without the stimuli as accurately as possible.

##### Reactivation (with stimuli)

The children had to recall sequences by tapping the key with the stimuli as accurately as possible. This task assessed reactivation resulting from reintroducing the stimuli (environmental model).

### Procedure

The experiment included several tasks, performed as follows:

#### Control Task: Synchronization With an Isochronous Sequence

The order of the auditory and visual modalities was counterbalanced between the participants for the isochronous synchronization tasks. Two trials were performed per modality.

#### Experimental Tasks

**FIGURE 2:**
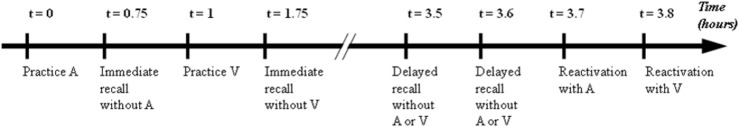
Tests of the experimental protocol : practice, immediate recall, differed recall and reactivation for auditory sequence (A) and visual sequence (V). Immediate retention corresponds to process between practice and immediate recall. Differed retention corresponds to process between immediate recall and differed recall (without stimuli). Reactivation corresponds to process between differed recall (without stimuli) and reactivation (with the reintroduction of the stimuli).

The children were required to perform the practice session using one of two modalities, followed by IMM, during which the metronome (i.e., visual or auditory sequence) was removed. The second modality was then practiced, followed by IMM. Two hours after the practice, both modalities were re-tested during DEL without the stimuli and in reactivation, during which the stimuli were reintroduced. During these 2 h, the children and their parents left for lunch. The order of the modalities (auditory or visual) and sequences (Sequence 1 or Sequence 2) was counterbalanced between the participants (Figure 2). Therefore, one participant learned Sequence 1 with auditory stimuli and Sequence 2 with visual stimuli whereas another participant learned Sequence 1 with visual stimuli and Sequence 2 with auditory stimuli.

##### Practice

**FIGURE 3:**
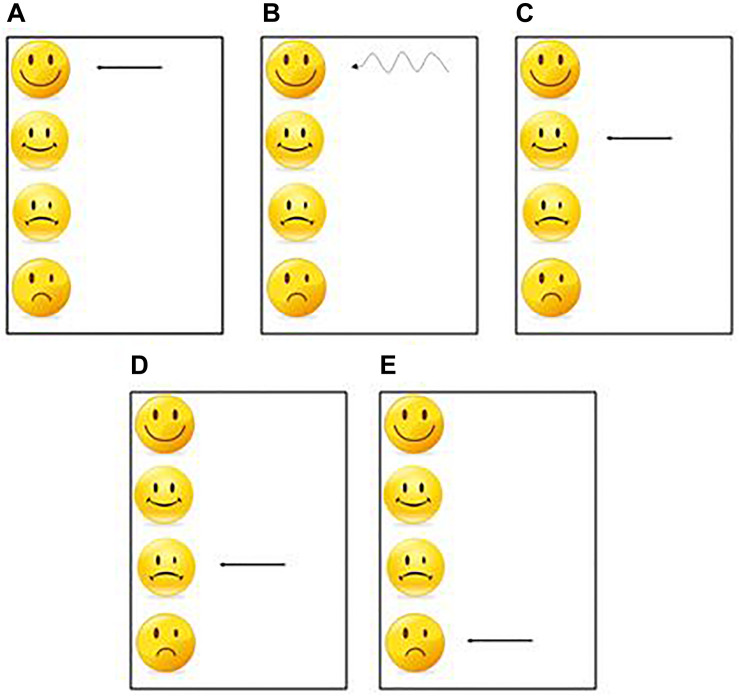
One of these 5 feedbacks was presented at the end of each practice trial. **(A)** The first feedback was displayed when the participant had an average synchronization between −20 ms and +20 ms and a standard deviation less than 20 ms. **(B)** The second feedback was displayed when the participant had an average synchronization between −20 ms and +20 ms and a standard deviation greater than 20 ms. **(C)** The 3^rd^ feedback was displayed when the participant had an average synchronization between −40 ms and −20 ms or between +20 and +40 ms. **(D)** The 4^th^ feedback was displayed when the participant had an average synchronization between −60 ms and −40 ms or between +40 ms and +60 ms. **(E)** The 5^th^ feedback was displayed when the participant had an average synchronization less than −60 ms or greater than 60 ms.

For each sequence, one per modality, each participant had 30 practice trials to learn the sequence. At the end of each trial a visual feedback was presented to the participants as a smiley face, indicating performance (Figure 3). This task corresponded to the learning phase and was used to test the effect of the practice.

##### Immediate recall

Immediately after the practice phase, the participants had three trials to recall the sequence without the stimuli. They performed the visual sequence without any stimuli immediately after practicing the visual sequence, and performed the immediate auditory recall without any stimuli immediately after practicing the auditory sequence. No feedback was given. They started when they wanted and stopped when they thought they had finished the sequence. After a 10-min break, the participants practiced the other sequence using the other modality [30 trials in the practice session + three IMM trials].

##### Delayed recall

Two hours after the practice session, the participants had to recall both sequences (three trials per sequence) without the stimuli. The order of the sequences was free. Children did not verbalize the number of the sequence but labeled the sequence “visual/square” or “auditory/tones” before starting. They started when they wanted and stopped when they thought they had finished the sequence. No feedback was given. Please note that seven TD children (35%) and five children with DCD (41.6%) were not able to reproduce the DEL sequences.

##### Reactivation

Participants had to produce the two sequences (three trials per sequence) with the stimuli in the same order as the practice. No feedback was given. Unlike DEL without a stimulus, this task was used to test reactivation by reintroducing the metronome.

### Data Analysis

#### Practice and Reactivation

Asynchrony and stability were calculated via a circular data processing method (Fisher, 1995) using CircStat. CircStat is a MATLAB Toolbox (MATLAB version 2015a) for circular statistics (Berens, 2009), recommended for cyclical data, particularly suited to synchronization data and sensitive to individual differences (Dalla Bella and Sowiński, 2015). Circular data processing has been used in the literature during manual tapping on a synchronization task for an isochronous rhythmic sequence in healthy adults (Sowiński and Dalla Bella, 2013), children with or without a neuro-developmental disorder (Puyjarinet et al., 2017) and patients with neurodegenerative diseases (Martin et al., 2017).

Processing involves representing each finger tap with an angle (unitary vector) on a 360° polar scale, where the circle represents the inter-beat interval of the stimuli. The resultant angle of vector *R* represents synchronization accuracy (Sowiński and Dalla Bella, 2013; Dalla Bella et al., 2017). For each subject and each trial, we obtained a resultant vector angle that we transformed into an absolute value (in order to average the data across the trials). Subsequently, the vector angles were converted into a percentage of asynchrony to obtain data on a linear scale and for better understanding. To give an example of conversion: an angle of 0° was converted to 0% asynchrony and an angle of 180° was converted to 50% asynchrony. The higher the percentage, the lower the synchronization. The length of resultant vector *R* (Sowiński and Dalla Bella, 2013; Dalla Bella et al., 2017) is referred to as synchronization stability and varies from 0 to 1: 0 corresponds to a uniform and random distribution of responses around the circle while 1 corresponds to a uniform distribution of responses in one direction. In other words, the longer the vector length is close to 1, the greater the stability for the synchronization of responses within the trials. The vector angle and the vector length were obtained via circular statistics using CircStat (Berens, 2009) in MATLAB, based on temporal tapping data.

For each subject and each trial, we obtained a percentage of absolute asynchrony representing accuracy and a vector length representing the stability of sensorimotor coordination synchronization. Please note that the first response was never taken into account in data processing because it was considered a “warm-up” step. Every three consecutive trials of the 30 practice trials were averaged to obtain 10 blocks of three practice trials for absolute asynchrony and vector length.

#### Immediate Recall and Delayed Recall (Without Stimuli)

These recalls without stimuli led to other analyses because (1) we recorded tap time only (with no stimulus) and (2) the participants started when they wanted, so the first interval was extremely variable from one individual to another.

The first tap had to align with 0° (as the first response was synchronized with the first imaginative stimulus). The first tap time was subtracted from all other times further to the responses so the first tap was well aligned with 0° and the following taps were in tempo with what the participant had done.

Between the responses, we added an accumulatively increasing stimuli time by putting the first 0° stimulus aligned with the first dummy response. From there, we performed the same data processing as the practice and reactivation sessions in order to obtain the vector angle and vector length of the three trials. The angle values of the three tests were highlighted as an absolute value so as to be able to average them. Finally, the angle was converted into a percentage.

#### Control Task: Synchronization With an Isochronous Sequence

Asynchrony (accuracy) and vector length (stability) were calculated using a fixed inter-stimuli interval of 1,000 ms. The number of errors was computed because this appeared to be a potential learning deficit marker in DCD (Blais et al., 2017). Errors corresponded to the additional taps compared to what the rhythmic stimuli proposed.

### Statistics

Data normality was verified using the Kolmogorov–Smirnov test (*p* > .05). The homogeneity of variance was verified for each analysis of variance (ANOVA); *df* and *p*-values underwent Greenhouse–Geisser correction, if necessary.

For the control synchronization task using an isochronous metronome, statistical Group (2) × Modality (2) analyses of variance (ANOVAs) were carried out with repeated measures on Modality (Auditory; Visual), to compare the children with DCD with the TD children (controls) on asynchrony, vector length and number of errors (*p* < 0.05).

For the practice session, statistical Group (2) × Modality (2) × Block (10) ANOVAs were carried out with repeated measures on Modality (Auditory; Visual) and Block (B1–B10) on asynchrony, vector length and number of errors.

For immediate retention, statistical Group (2) × Modality (2) × Immediate Recall (2) ANOVAs were carried out with repeated measures on Modality (Auditory; Visual) and Immediate Recall (B10; IMM) for asynchrony, vector length and number of errors. Please note that we compared the last practice block (B10: mean of the last three practice trials) with the immediate retention block (IMM: mean of three retention trials).

For delayed retention, statistical Group (2) × Modality (2) × Recall (2) ANOVAs were carried out with repeated measures on Modality (Auditory; Visual) and Recall (IMM; DEL) for asynchrony, vector length and number of errors.

For reactivation, statistical Group (2) × Modality (2) × Reactivation (2) ANOVAs were carried out with repeated measures on Modality (Auditory; Visual) and Reactivation (DEL; REAC) for asynchrony, vector length and number of errors.

The *p*-value was fixed at *p* < 0.05 for each analysis. η^2^ was reported for significant effects on the ANOVA. Separate *post hoc t*-tests were computed for independent groups using a Bonferroni correction for multiple comparisons.

## Results

### Control Task: Synchronization With an Isochronous Sequence

#### Asynchrony (Accuracy)

The ANOVA revealed a main Group effect on asynchrony [*F*(1,30) = 7.496, *p* = 0.01; η^2^ = 0.199]. Asynchrony was higher in the DCD group (12.4% ± 7.2) than the control group (6.9% ± 4.2) irrespective of Modality, reflecting a lower synchronization accuracy in children with DCD than the control children.

#### Vector Length (Stability)

The ANOVA revealed a Group effect on vector length [*F*(1,30) = 12.881, *p* = 0.001; η^2^ = 0.184]. Vector length was lower in the DCD group (0.675 ± 0.112) than the control group (0.805 ± 0.090) irrespective of Modality, reflecting lower synchronization stability in children with DCD than the control children.

The ANOVA revealed a Modality effect on vector length [*F*(1,30) = 8.508, *p* = 0.006; η^2^ = 0.008]. Vector length was higher in the auditory Modality (0.801 ± 0.147) than the visual Modality (0.711 ± 0.137) irrespective of the Group, reflecting higher synchronization stability in the auditory modality than the visual modality for both groups.

#### Number of Errors

The ANOVA revealed a Group effect on the number of errors [*F*(1,30) = 5.993, *p* = 0.020; η^2^ = 0.166]. The number of errors was higher in the DCD group (1.645 ± 0.950) than the control group (1.078 ± 0.334) irrespective of the Modality.

### Experimental Task: Learning Non-isochronous Sequences

#### B1–B10: Practice Effect

##### Asynchrony (accuracy)

The ANOVA revealed a main Group effect on asynchrony [*F*(1,30) = 5.682, *p* = 0.023; η^2^ = 0.008]. Asynchrony was higher in the DCD group (25.5% ± 10.3) than the control group (19.4% ± 10.8), irrespective of Modality and Block, reflecting lower synchronization accuracy in children with DCD than the control children.

The ANOVA revealed a main Block effect on asynchrony [*F*(9,270) = 20.511, *p* < 0.001; η^2^ = 0.116]. Asynchrony was higher during Block 1 (29.8% ± 8.2) than Block 10 (16.7% ± 10.7) [*t*(30) = 8.897; *p* = 6.45 10^–10^) irrespective of the Group and Modality, suggesting increased accuracy with practice for both groups.

**FIGURE 4:**
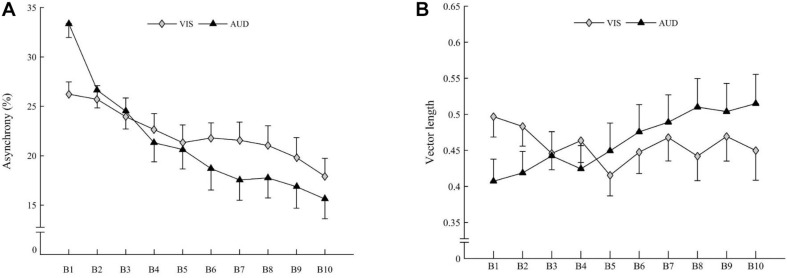
**(A)** Mean asynchrony of children (both groups averaged) for visual modality (gray diamonds) and auditory modality (black triangles). **(B)** Mean vector length of children (both groups averaged) for visual modality (gray diamonds) and auditory modality (black triangles). Vertical bars represent inter-individual variability (standard error).

The ANOVA revealed Block × Modality interaction on asynchrony [*F*(9,270) = 4.080, *p* < 0.001; η^2^ = 0.022]. Irrespective of the Group, asynchrony decreased with the Block, most significantly in the auditory Modality (Figure 4A), suggesting that accuracy significantly increased with practice for both groups in the auditory Modality.

##### Vector length (stability)

The ANOVA revealed a Group effect on vector length [*F*(1,30) = 4.534, *p* = 0.041; η^2^ = 0.009]. The Vector length was lower in the DCD group (0.40 ± 0.18) than the control group (0.49 ± 0.18) irrespective of the Modality, reflecting lower synchronization stability in children with DCD than the control children.

The ANOVA revealed a Block × Modality interaction on vector length [*F*(9,270) = 3.002, *p* = 0.001; η^2^ = 0.026]. Irrespective of Group, the vector length increased with the Block for the auditory Modality [*t*(30) = 3.19; *p* = 0.003] but not for the visual Modality [*t*(30) = 1.55; *ns*], suggesting a stability increase with practice for both groups for the auditory Modality only (Figure 4B).

##### Number of errors

The ANOVA revealed a main Group effect on the Number of errors [*F*(1,30) = 6.213, *p* = 0.018; η^2^ = 0.122]. The Number of errors was higher in the DCD group (1.241 ± 1.217) than the control group (0.631 ± 0.766), irrespective of the Modality and Block.

The ANOVA revealed a Modality × Block interaction on the Number of errors [*F*(9,270) = 2.565, *p* = 0.007; η^2^ = 0.022]. Irrespective of the Group, the Number of errors decreased with the Block for the auditory Modality only [*t*(30) = 3.101; *p* = 0.004].

#### End of Practice (B10) vs Immediate Recall: Immediate Retention

##### Asynchrony (accuracy)

The ANOVA revealed a main Group effect on asynchrony [*F*(1,30) = 5.230, *p* = 0.029; η^2^ = 0.009]. The Vector angle was higher in the DCD group (23.6% ± 10.7) than the control group (17.8% ± 10.1) irrespective of the Modality and IMM, reflecting lower synchronization accuracy in children with DCD than the control children.

The ANOVA revealed an IMM effect on asynchrony [*F*(1,30) = 16.397, *p* < 0.001; η^2^ = 0.123]. Asynchrony was higher in IMM (23.2% ± 9.6) than at the end of the practice (B10) (16.7% ± 10.7) irrespective of the Group and Modality suggesting decreased synchronization accuracy for immediate retention, when stimuli were withdrawn.

##### Vector length (stability)

The ANOVA revealed an IMM effect on vector length [*F*(1,30) = 26.712, *p* < 0.001; η^2^ = 0.470]. The Vector length was lower in IMM (0.31 ± 0.11) than at the end of the practice (B10) (0.48 ± 0.23), suggesting that stability decreased when stimuli were withdrawn, irrespective of the Group and Modality.

##### Number of errors

The ANOVA revealed a main Group effect on the number of errors [*F*(1,30) = 9.651, *p* = 0.004; η^2^ = 0.174]. Irrespective of the IMM and the Modality, the number of errors was higher in the DCD group (1.43 ± 1.23) than the control group (0.57 ± 0.75).

The ANOVA revealed an IMM effect on the number of errors [*F*(1,30) = 17.252, *p* < 0.001; η^2^ = 0.005]. The Number of errors was higher during IMM (1.08 ± 1.16) than during the final block of the practice (B10) (0.71 ± 0.88) irrespective of the Group and Modality.

**FIGURE 5:**
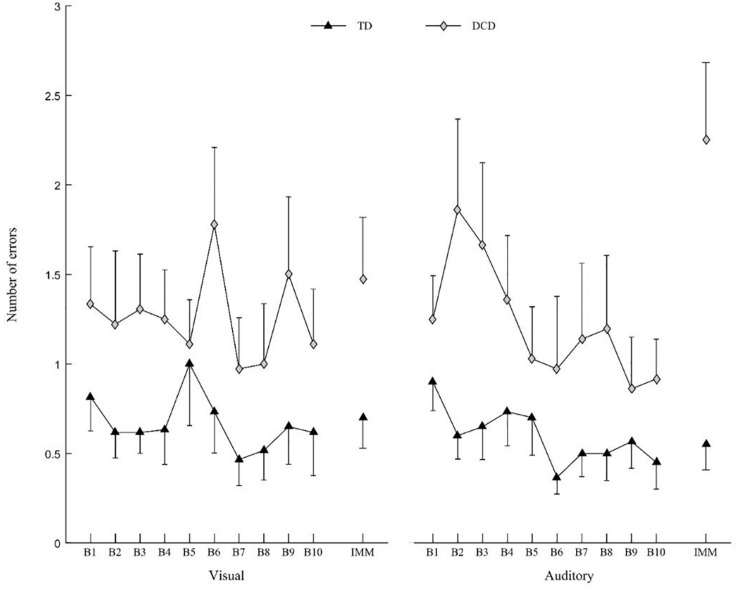
Mean number of errors for DCD group (gray diamonds) and TD group (black triangles) for visual (left) and auditory modality (right). Vertical bars represent inter-individual variability (standard error).

The ANOVA revealed a Group × IMM interaction [*F*(1,30) = 11.172; *p* = 0.002; η^2^ = 0.003], Modality × IMM interaction [*F*(1,30) = 5.627; *p* = 0.024; η^2^ = 0.014] and Group × Modality × IMM interaction on the number of errors [*F*(1,30) = 5.254; *p* = 0.029; η^2^ = 0.013]. For the auditory Modality only, the number of errors increased in the DCD group between the end of the practice (B10) (0.92 ± 0.76) and the IMM (2.25 ± 1.49) [*t*(30) = 3.844; *p <* 0.001], whereas for the visual modality, the number of errors did not increase between B10 (1.11 ± 1.06) and the IMM (1.47 ± 1.20) [*t*(30) = 1.65; *ns*] (Figure 5).

#### Immediate Recall vs Delayed Recall: Delayed Retention

##### Asynchrony (accuracy)

The ANOVA revealed no significant effect or interaction.

##### Vector length (stability)

The ANOVA revealed no significant effect or interaction.

##### Number of errors

The ANOVA revealed a main Group effect on the number of errors [*F*(1,18) = 8.41, *p* = 0.009; η^2^ = 0.318]. Irrespective of Modality and Recall phase, the number of errors was higher in the DCD group (1.48 ± 1.02) than the control group (0.61 ± 0.76).

#### Delayed Recall vs Reactivation: Reactivation

##### Asynchrony (accuracy)

The ANOVA revealed no significant effect or interaction.

##### Vector length (stability)

The ANOVA revealed a Reactivation of stimulus effect on vector length [*F*(1,18) = 50.333, *p* < 0.001; η^2^ = 0.736]. Irrespective of the Group and Modality, the vector length was higher (more stable) for Reactivation (0.54 ± 0.19) than DEL without a stimulus (0.27 ± 0.12).

##### Number of errors

The ANOVA revealed a main Group effect on the number of errors [*F*(1,18) = 10.994, *p* = 0.003; η^2^ = 0.212]. Irrespective of Modality and Reactivation, the number of errors was higher in the DCD group (1.23 ± 1.06) than the control group (0.52 ± 0.71).

The ANOVA revealed a Reactivation of stimulus effect on the number of errors [*F*(1,30) = 4.654, *p* = 0.044; η^2^ = 0.009]. Irrespective of the Group and Modality, the number of errors was higher for DEL without a stimulus (0.98 ± 1.01) than Reactivation (0.56 ± 0.75).

## Discussion

The purpose of this study was to test SMS and procedural learning for a sensorimotor temporal sequence specified by auditory or visual stimuli in DCD. We predicted that children with DCD would have more difficulties synchronizing, learning, retaining, and reactivating a new temporal sensorimotor sequence than TD children. Moreover, we expected that difficulties would be modulated by the sensory modality of the stimuli, with a greater learning deficit for auditory than visual stimuli, as per the hypothesis of Trainor et al. (2018). Our results were partially consistent with our hypotheses.

Firstly, during the SMS task using isochronous stimuli, children with DCD demonstrated less accurate and stable synchrony than TD children for both auditory and visual stimuli. They also made more errors than their TD peers. Thus, our results indicate that an overarching synchronization deficit is present in DCD, regardless of the visual and auditory modality of the stimuli, as per previous findings on auditory stimuli (Williams et al., 1992; Whitall et al., 2008; Rosenblum and Regev, 2013; Puyjarinet et al., 2017) and auditory and visual stimuli (Whitall and Clark, 2018; Lê et al., 2020). Given that SMS was also impaired for both visual and auditory stimuli when children had to respond with verbal responses (Lê et al., 2020), it is possible that the general – effector-independent and modality-independent – deficit in SMS is possibly due to a deficit in timing perception in DCD, as proposed by Trainor et al. (2018).

Secondly, the DCD group was as able as the TD group in improving accuracy and stability and decreasing the number of errors with practice on the non-isochronous sequence, which challenges the idea that children with DCD do not use sensory information to improve performance (Whitall et al., 2006; Mackenzie et al., 2008; Roche et al., 2011). Therefore, learning a new temporal perceptual-motor sequence seems to be retained in children with DCD. These results are in line with previous results showing that learning is relatively preserved in DCD (Wilson et al., 2003; Blais et al., 2018; Lê et al., 2020) and challenges the procedural learning deficit hypothesis postulated by Nicolson and Fawcett (2007).

Thirdly, regarding the effect of the sensory modality, we found a more significant improvement in temporal accuracy and stability during practice with auditory compared to visual stimuli in TD children and children with DCD. This result suggests that children with or without DCD benefit more from auditory stimuli than visual stimuli when learning a temporal sequence. However, the benefit of auditory stimuli seems to be transient for children with DCD, who demonstrated a significant increase in errors immediately after the removal of the auditory stimuli (IMM) and after a delay (DEL). The new increase in performance with the reintroduction of the auditory stimuli (reactivation test) suggests that children with DCD have a specific deficit in terms of integrating an audio-motor sequence in their memory. Given that the recall and reactivation tests involved withdrawing and reintroducing environmental information specifying the audio-motor sequence, the modulation of performance in children with DCD suggests that the auditory information provides a guidance effect (Salmoni et al., 1984; Walter and Swinnen, 1994). In other words, children with DCD depend on environmental auditory information during practice. This dependency, which is specific to auditory information, suggests that the auditory information results in the establishment of a perception-action coupling in DCD, as already suggested in Lê et al. (2020). Children with DCD fail to properly reproduce the temporal sensorimotor sequence by themselves once the auditory information is removed. Children with DCD may be able to transiently adapt to environmental stimuli when present, but are not able to really integrate the temporal sequence in their memory. Another view is that auditory stimuli are so attractive that, when withdrawn, children are prone to making more errors than with visual stimuli, for which withdrawal does not result in as much disruption (Repp and Penel, 2004; Repp and Su, 2013; Thaut, 2015). In this case, our results suggest for the first time that auditory stimuli are more attractive than visual stimuli in DCD children when compared with TD children.

In short, TD children benefited from auditory information at each stage of practice, retention, and reactivation, contrary to DCD children, who benefited from auditory information during practice and reactivation (when the stimuli were present) but not for retention (when the stimuli were removed and the sequence had to be produced from memory). For the first time, these results demonstrate the superiority of the auditory modality from SMS to the procedural learning of a new sensorimotor temporal sequence in TD children. As per the literature, in healthy adults (Repp and Penel, 2002; Chauvigné et al., 2014; Merchant et al., 2015; Iversen and Balasubramaniam, 2016), it is possible for common cerebral structures to underlie both SMS and temporal sequence learning with auditory stimuli. In DCD, even if the auditory information helps improve performance during practice and reactivation (with stimuli), it does not help retention (without stimuli). Therefore, our results are partially in line with the proposal by Trainor et al. (2018), who hypothesized that “motor control of children with DCD would benefit from the addition of rhythmic auditory cues” (Trainor et al., 2018). Our results actually led us to conclude that visual stimuli are more likely to improve the learning and memorization of temporal motor sequences in children with DCD. This result adds to the previous findings of Lê et al. (2020), showing that visual information could be a more appropriate cue for the long-term retention of temporal sequences.

## Limits and Prospects

A few limits and prospects can be mentioned for this study. Firstly, given that each child had to learn and retain two temporal sensorimotor sequences, an interference effect may have taken place. As previously explained by Schmidt and Young (1987), when two similar tasks are practiced sequentially, they may interfere each other. Such a phenomenon may have occurred in our study, but we could not investigate it given that learning of the two tasks was counterbalanced. However, it would be interesting to study the role of interference in DCD in the future.

Another limitation of our study is that we did not test the perceptual discrimination abilities of the participants. Errors in sensorimotor synchronization may have resulted from a deficit in timing perception, in line with the recent assumption of Trainor et al. (2018). Trainor et al. (2018) hypothesized that auditory perceptual timing deficits may be core characteristics of DCD, but no studies have yet demonstrated this assumption (Trainor et al., 2018). On the other hand, please note that errors correspond to additional responses (more taps than required). This result could be a marker of a motor inhibition deficit in DCD, as reported in a previous study about learning in teenagers with DCD (Blais et al., 2017, 2018). Therefore, we cannot be sure that errors identified in this study were due to a deficit in (perceptual) processing of the stimuli or a deficit (inhibition) in motor output.

Moreover, there might be heterogeneity in the way children memorize the sequence, explaining why we cannot observe the effects of DEL for instance. It is also difficult to explain the high incidence of children (both TD and DCD) who were unable to reproduce the DEL sequence. In the future, it may be interesting to study individual strategies that could give information on specific processes at stake in the learning and memorization of temporal sequences.

Another limitation is the sample size of our study, with only 12 participants with DCD. However, the exclusion of comorbidities and the restricted inclusion criteria (with a M-ABC score below the 5th percentile) were a real advantage for this study.

Finally, our results open prospects for studying the cerebral correlates of learning in DCD. The model of Doyon and Benali (2005) suggests that sequence learning is supposed to involve the cortico-striato-cortical loop, whereas the cortico-cerebello-cortical loop is involved in sensorimotor adaptation. On this basis, it seems that the cortico-striato-cortical loop could be altered in DCD (Cignetti et al., 2020; Tallet and Wilson, 2020). In this study, we evaluated learning with only 30 practice trials, which corresponds to the fast-learning stage according to Doyon and Benali (2005). This stage involves a large cerebral network, including not only the striatum but also a set of structures such as the cerebellum, motor cortical regions, parietal, prefrontal, and limbic regions. In the future, neural functional connectivity measurements to and from the striatum may be good way to understand the relationships between observable behavior and cerebral indices (Blecher et al., 2016). Studying learning at the slow learning stage (specifically involving the cortico-striatal network) would certainly show additional results on motor learning and memory in children with DCD.

To date, no intervention studies have specifically tested whether children with DCD need more practice compared to TD children in order to reach a similar performance level in motor learning tasks (Schoemaker and Smits–Engelsman, 2015; Smits–Engelsman et al., 2018). In our study, the DCD children may have needed more practice compared to TD children to retain the audio-motor sequence. In other words, children with DCD may require longer to reach saturated learning for auditory stimuli.

All in all, our results encourage the continuation of research on aspects involving procedural memory and neural correlates in DCD to be considered as necessary.

## Data Availability Statement

The raw data supporting the conclusions of this article will be made available by the authors, without undue reservation.

## Ethics Statement

The studies involving human participants were reviewed and approved by the National Ethical Committee of the Institute for Medical Research (Inserm, 2014-AO1239-38). Written informed consent to participate in this study was provided by the participants’ legal guardian/next of kin.

## Author Contributions

YC, J-MA, MJ, and JT conceived the project and obtained the financial support for this experimentation. MB and JT conceived and planned the experiment, analyzed the results, and wrote the manuscript. MB and SM carried out the experiment. All authors provided critical feedbacks on the manuscript.

## Conflict of Interest

The authors declare that the research was conducted in the absence of any commercial or financial relationships that could be construed as a potential conflict of interest.

## Footnotes

1 www.neurobs.com
